# Metabolic patterns on [^18^F]FDG PET/CT in patients with unresectable stage III NSCLC undergoing chemoradiotherapy ± durvalumab maintenance treatment

**DOI:** 10.1007/s00259-023-06192-6

**Published:** 2023-03-23

**Authors:** Adrien Holzgreve, Julian Taugner, Lukas Käsmann, Philipp Müller, Amanda Tufman, Niels Reinmuth, Minglun Li, Michael Winkelmann, Lena M. Unterrainer, Alexander E. Nieto, Peter Bartenstein, Wolfgang G. Kunz, Jens Ricke, Claus Belka, Chukwuka Eze, Marcus Unterrainer, Farkhad Manapov

**Affiliations:** 1 Department of Nuclear Medicine, University Hospital, LMU Munich, Marchioninistr. 15, 81377 Munich, Germany; 2 Department of Radiation Oncology, University Hospital, LMU Munich, Munich, Germany; 3 Member of the German Center for Lung Research (DZL), Comprehensive Pneumology Center Munich (CPC-M), Munich, Germany; 4 German Cancer Consortium (DKTK), Partner Site Munich, Munich, Germany; 5 Department of Radiology, University Hospital, LMU Munich, Munich, Germany; 6 Department of Internal Medicine V, University Hospital, LMU Munich, Munich, Germany; 7 Asklepios Lung Clinic, Munich‑Gauting, Germany

**Keywords:** Non-small-cell lung cancer (NSCLC), [^18^F]FDG PET/CT, Durvalumab immune checkpoint inhibitor consolidation, Durvalumab maintenance treatment, Immunotherapy-related adverse events (irAE)

## Abstract

**Purpose:**

In patients with unresectable stage III non-small-cell lung cancer (NSCLC), durvalumab maintenance treatment after chemoradiotherapy (CRT) significantly improves survival. So far, however, metabolic changes of tumoral lesions and secondary lymphoid organs under durvalumab are unknown. Hence, we assessed changes on [^18^F]FDG PET/CT in comparison to patients undergoing CRT alone.

**Methods:**

Forty-three patients with [^18^F]FDG PET/CT both before and after standard CRT for unresectable stage III NSCLC were included, in 16/43 patients durvalumab maintenance treatment was initiated (CRT-IO) prior to the second PET/CT. Uptake of tumor sites and secondary lymphoid organs was compared between CRT and CRT-IO. Also, readers were blinded for durvalumab administration and reviewed scans for findings suspicious for immunotherapy-related adverse events (irAE).

**Results:**

Initial uptake characteristics were comparable. However, under durvalumab, diverging metabolic patterns were noted: There was a significantly higher reduction of tumoral uptake intensity in CRT-IO compared to CRT, e.g. median decrease of SUV_max_ –70.0% vs. –24.8%, *p* = 0.009. In contrast, the spleen uptake increased in CRT-IO while it dropped in CRT (median + 12.5% vs. –4.4%, *p* = 0.029). Overall survival was significantly longer in CRT-IO compared to CRT with few events (progression/death) noted in CRT-IO. Findings suggestive of irAE were present on PET/CT more often in CRT-IO (12/16) compared to CRT (8/27 patients), *p* = 0.005.

**Conclusion:**

Durvalumab maintenance treatment after CRT leads to diverging tumoral metabolic changes, but also increases splenic metabolism and leads to a higher proportion of findings suggestive of irAE compared to patients without durvalumab. Due to significantly prolonged survival with durvalumab, survival analysis will be substantiated in correlation to metabolic changes as soon as more clinical events are present.

## Introduction

The groundbreaking results of the PACIFIC trial have recently led to a new standard of care in patients with unresectable stage III non-small-cell lung cancer (NSCLC), consisting of a now strong recommendation for durvalumab consolidation after chemoradiotherapy [1–3]. Although durvalumab is a monoclonal antibody directed against the programmed death-ligand 1 (PD-L1), even NSCLC patients with a rather low tumoral PD-L1 expression benefit from durvalumab and the follow-up data of the PACIFIC trial confirm the overwhelming survival benefit across various patients [4–7].

Yet, little is known about the metabolic uptake patterns on [^18^F]FDG PET/CT in NSCLC patients undergoing durvalumab maintenance treatment. [^18^F]FDG is sensitive to increased tumoral metabolism and therefore enables an apprehension of vital tumor tissue and its distribution in the body on PET beyond merely morphological changes [8, 9]. Thus, [^18^F]FDG PET/CT is a preferred standard imaging modality for the staging of NSCLC and, in addition to therapy response assessment, holds value for local treatment planning in the context of chemoradiotherapy [10, 11]. However, [^18^F]FDG lacks specificity for vital tumor tissue while also being sensitive to diverse (auto-) inflammatory and reactive processes, such as present in the case of immunotherapy-related adverse events (irAE) [12]. Indeed, in the current era of evolving immunotherapeutic drugs including check point inhibitors such as durvalumab, several related imaging findings might be confounded as vital tumor and, for instance, hamper early therapy response assessment in NSCLC patients [13]. Also, diverging uptake characteristics inherent to the tumoral lesions under immunotherapy must be taken into account [14].

Hence, we here assessed metabolic changes of tumoral lesions and secondary lymphoid organs on [^18^F]FDG PET/CT in a homogenous cohort of patients with unresectable stage III NSCLC after durvalumab initiation in comparison to matched control patients undergoing CRT alone.

## Methods

### Patients

Forty-three patients with locally advanced, unresectable NSCLC stage IIIA–C in good functional condition (ECOG ≤ 1) and with available [^18^F]FDG PET/CT both before the initiation and after the completion of standard CRT were retrospectively included at a single tertiary cancer center. In 16/43 patients, durvalumab maintenance treatment was initiated in between the end of CRT and the second PET/CT (CRT-IO); the other 27/43 patients represent a matched control group (CRT). Minor parts of the CRT-IO cases were subject to prior separate analyses with a different scope [14]. All cases were histologically verified and (re-)classified according to the current 2018 UICC 8^th^ edition. All patients received cranial contrast-enhanced magnetic resonance imaging (MRI) or computed tomography scan (CT). Routine blood work to assess kidney function and complete blood count as well as pulmonary function testing was performed in all cases, patients with poor lung function either on regular spirometry (FEV1 < 1 l) or on diffusion capacity testing (D_LCO_ < 40%) and patients receiving long-term oxygen therapy were excluded from this study. Status of unresectability and treatment decisions were in all cases based on the recommendation of a multidisciplinary tumor board including experienced oncologists, pulmonologists, thoracic surgeons, and radiation oncologists.

16/43 patients received additional durvalumab maintenance treatment up to 12 months (i.e., 24 cycles à 10 mg/kg intravenously every 2 weeks). Durvalumab administration was stopped in the case of tumor progression or when intolerable toxicity was met according to Version 5 of the Common Toxicity Criteria for Adverse Events (CTCAE).

### [^18^F]FDG PET/CT image acquisition

Acquisition of [^18^F]FDG PET/CT images was performed as previously described using imaging parameters as jointly recommend by the European Association of Nuclear Medicine (EANM), the Society of Nuclear Medicine and Molecular Imaging (SNMMI) and the European Society for Radiotherapy and Oncology (ESTRO) [10, 14, 15]. In brief, whole-body or torso PET image acquisition was started in the treatment position on a carbon fiber couch approx. 60 min after the administration of [^18^F]FDG and, whenever possible, of 10–20 mg furosemide and 10–20 mg butylscopolamine using a GE Discovery 690 PET/CT scanner (GE HealthCare, Chicago, IL, USA). PET imaging reconstruction was performed as previously described [14]. SUV quantification was based on total body weight. PET imaging was simultaneously acquired with a diagnostic CT scan after the administration of 350 mg of iomeprol (Imeron®) at 1.5 ml/kg body weight (portal-venous phase). All scans were performed at the same institution.

### Data evaluation

Two experienced nuclear medicine physicians / radiologists evaluated the [^18^F]FDG PET/CT images. The maximum standardized uptake value (SUV_max_) of tumoral lesions was assessed. Mean activity of the spleen (SUV_spleen_) and the bone marrow (SUV_BM_) were derived using a 3.0 cm and a 1.5 cm spherical VOI placed in the center of the spleen or in lumbar vertebrae, respectively. Here, the mean uptake of L4 and L5 served as bone marrow uptake; other lumbar vertebrae were only used in case of relevant (degenerative) changes in L4 and L5, e.g. compression fracture. Short and long axis diameter of tumor manifestations were measured on CT images. For estimation of the spleen volume, a three-dimensional approach using the product of the spleen length, maximal width, and thickness on CT images was used [16].

Moreover, readers were blinded for the administration of durvalumab and scans were reviewed for findings suspicious for immunotherapy-related adverse events (irAE).

All imaging data were evaluated using the Hybrid Viewer 3D software (Hermes Medical Solutions, Stockholm, Sweden).

### Clinical parameters, outcome parameters, tumor progression

In addition to [^18^F]FDG PET/CT imaging parameters, patient characteristics were collected and included patients’ age, sex, tumor subtype according to histology, and UICC stage (IIIA-C). Progression-free survival (PFS) was defined according to RECIST 1.1 [17–19]. Overall survival (OS) was defined as the time from primary diagnosis until death. All [^18^F]FDG PET/CT-derived and further clinical parameters were correlated to PFS and OS.

### Statistics

IBM SPSS Statistics was used for the statistical analysis (Version 26, IBM, Armonk, NY, USA). Descriptive statistics of continuous parameters are given as the median (range). The Shapiro–Wilk test was used to check for normal distribution. The Wilcoxon signed-rank test and the Mann–Whitney U were used to investigate group differences in dependent and independent, not-normally distributed continuous parameters, respectively. Linear bivariate associations were assessed using Pearson’s correlation. Continuous clinical and [^18^F]FDG PET/CT-derived parameters underwent median split dichotomization for survival analysis. The Kaplan–Meier estimator and log-rank test were applied for survival analysis of PFS and OS (results given as median survival with 95% confidence interval, CI). A two-tailed p-value < 0.05 was considered as statistically significant.

## Results

### Patient characteristics

In the overall group, median age was 67.5 years (33.6 – 76.9), 14/43 patients were female (32.6%). Patients receiving durvalumab maintenance treatment did not significantly differ from patients undergoing chemoradiotherapy alone regarding age (*p* = 0.090), sex (*p* = 0.889), tumor type according to histology (*p* = 0.583) and UICC stage (*p* = 0.594). The patient characteristics are displayed in Table 1.

**Table 1:** Patient characteristics and quantitative [^18^F]FDG PET/CT results

	Overall Group (n = 43)	CRT-IO (n = 16)	CRT (n = 27)	Significance
Age				
Median (range) in years	67.5 (33.6 – 76.9)	66.5 (43.8 – 72.9)	67.7 (33.6 – 76.9)	*p* = 0.090
Sex				
Male	29 (67.4%)	11 (68.8%)	18 (66.7%)	*p* = 0.889
Female	14 (32.6%)	5 (31.3%)	9 (33.3%)	
Histology				
Adenocarcinoma	19 (44.2%)	8 (50.0%)	11 (40.7%)	*p* = 0.583
Squamous cell carcinoma	18 (41.9%)	6 (37.5%)	12 (44.4%)	
other	6 (14.0%)	2 (12.5%)	4 (14.8%)	
Stage				
III A	14 (32.6%)	5 (31.3%)	9 (33.3%)	*p* = 0.594
III B	14 (32.6%)	7 (43.8%)	7 (25.9%)	
III C	15 (34.9%)	4 (25.0%)	11 (40.7%)	
COPD	14 (32.6%)	5 (34.9%)	9 (33.3%)	*p* = 0.889
Induction chemotherapy				
Yes	19 (44.2%)	7 (43.8%)	12 (44.4%)	*p* = 0.965
No	24 (55.8%)	9 (56.2%)	15 (55.6%)	
Survival months (95% CI)				
PFS	8.0 (4.8 – 11.2)	19.0 (n/a)	6.0 (2.6 – 9.4)	*p* = 0.008
OS	50.0 (18.6 – 81.4)	Median not reached	26.0 (17.5 – 34.5)	*p* = 0.004
PET/CT 1 median (range)				
Tumor, SUV_max_	13.2 (2.1 – 42.8)	13.4 (4.0 – 42.8)	12.6 (2.1 – 24.9)	*p* = 0.439
Bone marrow, SUV_mean_	1.8 (1.1 – 7.7)	1.4 (1.1 – 2.4)	1.8 (1.1 – 7.7)	*p* = 0.022
Spleen, SUV_mean_	2.1 (1.4 – 3.5)	2.1 (1.5 – 2.6)	2.2 (1.4 – 3.5)	*p* = 0.792
Spleen, volume in ml	121.2 (36.6 – 266.4)	104.8 (36.6 – 177.9)	135.7 (50.0 – 266.4)	*p* = 0.138
PET/CT 2 median (range)				
Tumor, SUV_max_	7.2 (1.5 – 23.7)	6.4 (3.7 – 8.7)	8.9 (1.5 – 23.7)	*p* = 0.015
Bone marrow, SUV_mean_	1.6 (1.1 – 2.9)	1.6 (1.1 – 2.8)	1.7 (1.1 – 2.9)	*p* = 0.272
Spleen, SUV_mean_	2.2 (1.1 – 3.1)	2.3 (1.5 – 3.1)	2.1 (1.1 – 3.1)	*p* = 0.085
Spleen, volume in ml	104.4 (47.6 – 261.3)	104.4 (47.6 – 187.9)	110.6 (52.1 – 261.3)	*p* = 0.453
PET/CT2–PET/CT1				
Delta in % (range)				
Tumor uptake	-36.9 (-88.3 – + 74.4)	-70.0 (-88.3 – + 72.8)	-24.8 (-77.8 – + 74.4)	*p* = 0.009
Bone marrow uptake	+ 3.7 (-74.6 – + 66.1)	+ 6.0 (-44.2 – + 66.1)	-3.0 (-74.6 – + 64.4)	*p* = 0.353
Spleen uptake	+ 2.2 (-39.3 – + 50.0)	+ 12.5 (-30.7 – + 50.0)	-4.4 (-39.3 – + 48.0)	*p* = 0.029
Spleen volume	-0.15 (-35.5 – + 68.6)	+ 14.1 (-35.5 – + 68.6)	-1.3 (-35.2 – + 36.8)	*p* = 0.092

The median time span between the first PET and the initiation of CRT was 0.8 months (IQR, 0.5–1.3 mo), the time span was slightly but significantly larger in CRT-IO vs. CRT alone (1.5 vs. 0.8 mo, p = 0.006). The median time span between the termination of CRT and the second PET was 3.1 months (IQR 2.0–5.0 mo) and it was slightly longer in CRT-IO compared to CRT alone (3.7 vs. 2.5 mo, p = 0.015). The median time span between the first PET and the second PET was 5.0 months (IQR 4.0–7.7 mo). In the CRT-IO group, the second PET took place 2.9 months (IQR 2.4–5.0 mo) after the initiation of durvalumab maintenance treatment.

### Quantitative parameters assessed on [^18^F]FDG PET/CT

The tumoral lesions showed markedly increased glucose metabolism before the start of chemoradiotherapy (SUV_max_ 13.2 in the overall group) and the tumoral uptake intensity did not differ in between the CRT-IO and the CRT groups before durvalumab initiation (SUV_max_ 13.4 vs. 12.6, *p* = 0.439). The splenic uptake initially was comparable in both groups (SUV_mean_ 2.1 vs. 2.1, *p* = 0.792), whereas the bone marrow uptake was slightly but significantly lower in the CRT-IO group (SUV_mean_ 1.4 vs. 1.8, *p* = 0.022). There were no significant differences in spleen volumes in the two groups before durvalumab initiation (104.8 ml vs. 135.7 ml, *p* = 0.138).

After the completion of chemoradiotherapy and after durvalumab initiation, however, diverging metabolic patterns were noted in dependency of the durvalumab administration: A significantly higher reduction of tumoral uptake intensity was noted in the CRT-IO group compared to the CRT group, e.g. decrease of SUV_max_ –70.0% vs. –24.8%, *p* = 0.009. The spleen uptake, in contrast, increased in the CRT-IO group and dropped in the CRT group (median + 12.5% vs. –4.4%, *p* = 0.029). This was in line with a trend towards an increasing spleen volume in the CRT-IO group as opposed to a decreasing spleen volume in the CRT alone group (+ 14.1% vs. – 1.3%, *p* = 0.092) without reaching the level of significance. No diverging uptake was noted in the bone marrow (+ 6.0% vs. –3.0, *p* = 0.353).

All results of the quantitative assessment of [^18^F]FDG PET/CT parameters are displayed in Table 1.

### Findings suggestive of irAE on [^18^F]FDG PET/CT

An increased rate of findings suggestive of irAE were noted in the CRT-IO group (12/16 patients, 75.0%) compared to the CRT group (8/27 patients, 29.6%), *p* = 0.005. Pneumonitis-typical imaging findings were the most common finding overall (in 27.9% of cases) as well as per group (CRT-IO: in 66.6%, CRT: in 14.8% of cases). Findings suggestive of gastritis, pleuritis, and sarcoid-like reaction were present in a higher proportion of patients of the CRT-IO group compared to the CRT group, and findings suggestive of thyroiditis, tonsillitis and vasculitis were only present in the CRT-IO group. Diagnostic findings suggestive of reactive changes in the colon occurred more frequently in the CRT group as compared to the CRT-IO group. Table 2 gives an overview of findings suggestive of an irAE on [^18^F]FDG PET/CT after durvalumab initiation in the CRT-IO group in comparison to CRT alone. Most suspected irAEs were either not or barely detectable using CT alone and instead primarily detected on the [^18^F]FDG PET imaging component. As far as documented in the patients’ history, a majority of those incidental findings were asymptomatic; most findings were not persistent on follow-up imaging in cases with available subsequent [^18^F]FDG PET/CT. In CRT-IO, only findings suggestive of pneumonitis in three cases and findings suggestive of gastritis in one case were persistent on the subsequent [^18^F]FDG PET/CT scan (available in 14/16 patients).

**Table 2:** Findings suggestive of irAE on [^18^F]FDG PET/CT

	Overall group (n = 43)	CRT-IO (n = 16)	CRT (n = 27)
Patients with ≥ 1 finding suspicious for an irAE			
CRT-IO vs. CRT, *p* = 0.005	20/43 (46.5%)	12/16 (75.0%)	8/27 (29.6%)
Type of suspected irAE			
Reactive changes in the colon	4 (9.3%)	1 (6.3%)	3 (11.1%)
Gastritis	3 (7.0%)	2 (12.5%)	1 (3.7%)
Pleuritis	3 (7.0%)	2 (12.5%)	1 (3.7%)
Pneumonitis	12 (27.9%)	8 (50.0%)	4 (14.8%)
Sarcoid-like reaction	2 (4.7%)	1 (6.3%)	1 (3.7%)
Thyroiditis	2 (4.7%)	2 (12.5%)	-
Tonsillitis	1 (2.3%)	1 (6.3%)	-
Vasculitis	1 (2.3%)	1 (6.3%)	-

**Fig. 1:**
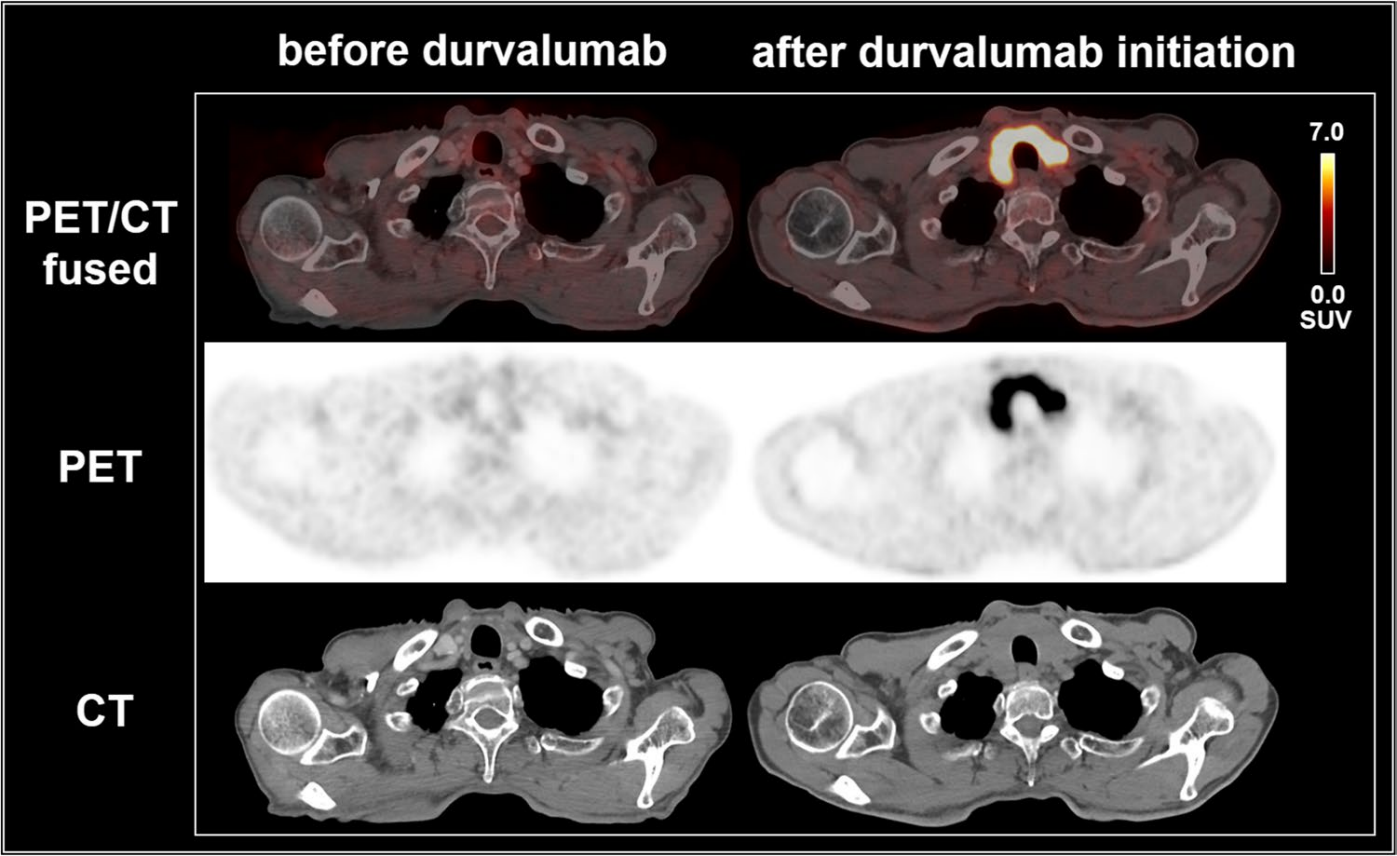
[^18^F]FDG PET/CT scans of a 69-year old man with stage IIIB NSCLC of the right upper lobe, cT3 cN2 cM0, adenocarcinoma, CK7-positive, TTF1-positive, PD-L1: 10%, active smoker (75 py), undergoing CRT with 63.6 Gy, Cisplatin/Vinorelbine, and durvalumab maintenance treatment, who developed an immunotherapy-related thyroiditis. After durvalumab administration, the thyroid gland significantly increased in volume and showed a markedly increased glucose metabolism on [^18^F]FDG PET/CT (SUV_max_ 17.0). The blood tests substantiated the suspicion of an immunotherapy-related thyroiditis with hyperthyroidism (TSH < 0.01 µU/ml) at the day of the PET/CT scan which turned to hypothyroidism 6 weeks later (TSH 56.4 µU/ml, fT4 < 0.3 ng/dl, fT3 < 1.0 pg/ml). During the follow-up time of 24 months the patient did not experience tumor progression

**Fig. 2:**
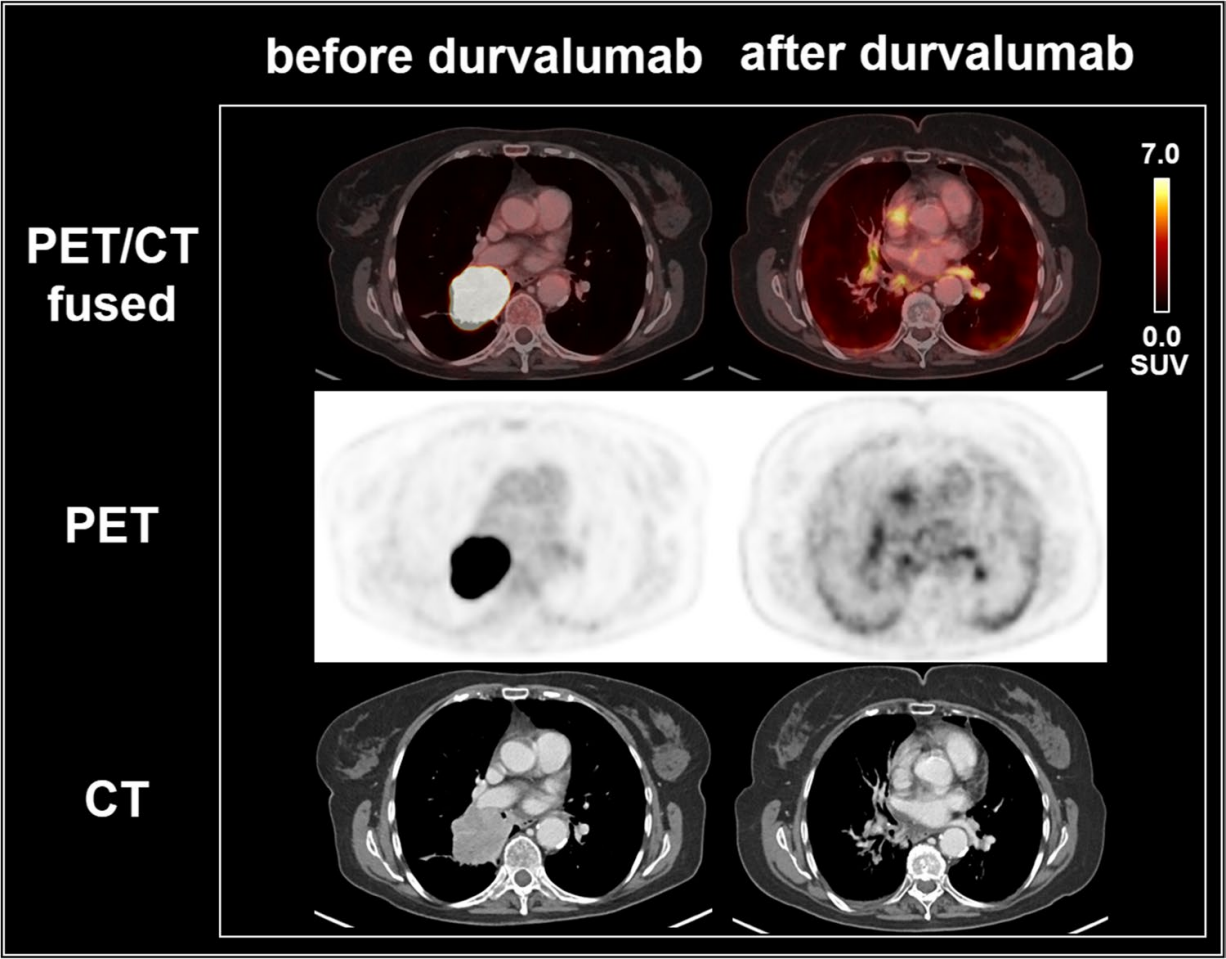
[^18^F]FDG PET/CT scans of a 71-year old woman with stage IIIB NSCLC of the right upper lobe, cT3 cN2 cM0, adenocarcinoma, ALK-negative, PD-L1: 40%, former smoker (30 py), undergoing CRT with 63.6 Gy, Cisplatin/Vinorelbine, and durvalumab maintenance treatment, who developed findings suggestive of an immunotherapy-related sarcoid-like reaction. After durvalumab administration, mediastinal and hilar lymph nodes were slightly enlarged and showed an increased uptake on [^18^F]FDG PET/CT (SUV_max_ 9.0). Follow-up imaging 3.5 months later revealed a decreasing size and a strongly decreasing, now normalized glucose metabolism of the thoracic lymph nodes, consistent with a prior immunotherapy-related sarcoid-like reaction. The patient had a PFS of 11 months and was still alive at the last follow-up after 34 months

**Fig. 3:**
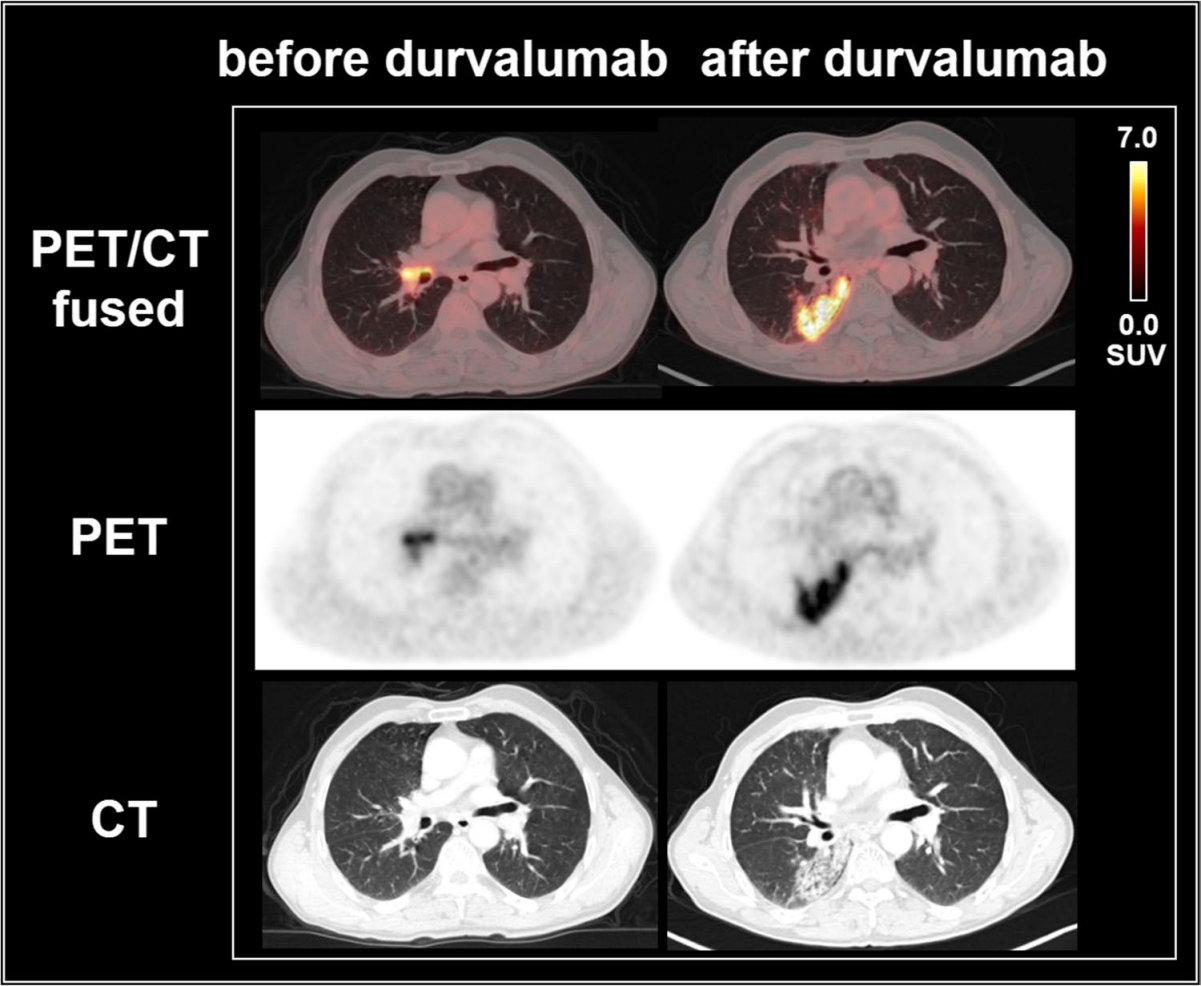
[^18^F]FDG PET/CT scans of a 62-year old man with stage IIIB NSCLC, cT3 cN2 cM0, squamous cell carcinoma, p40-positive, PD-L1: 80%, former smoker (70 py), undergoing CRT with 63.6 Gy, Cisplatin/Vinorelbine, and durvalumab maintenance treatment, who developed pneumonitis-typical imaging findings. The latter occurred in both groups, most likely also as a direct effect of irradiation. After durvalumab administration, increased reticular and ground-glass opacities and new patchy consolidations predominantly in the right lower lobe, were observed. These correlated markedly with increased glucose uptake as assessed by [^18^F]FDG PET/CT (SUVmax 8.1). Although the metabolism at the next available [^18^F]FDG PET/CT 7 months later had returned to near normal, the CT alterations only slowly regressed and small residuals were still present on the imaging at the last follow up after 24 months; the patient did not experience tumor progression

Figure 1 gives a pictorial example of a patient with an immunotherapy-related thyroiditis. Figure 2 gives a pictorial example of a patient who developed findings suggestive of an immunotherapy-related sarcoid-like reaction. Figure 3 gives a pictorial example of a patient who developed findings suggestive of a pneumonitis. Pneumonitis-typical imaging findings occurred in both the CRT-IO group and the CRT alone group, most likely as a direct effect of irradiation. Chronic obstructive pulmonary disease (COPD), as a factor influencing the course of pneumonitis, was equally distributed in both groups (*p* = 0.889).

### Survival analysis in correlation to [^18^F]FDG PET/CT findings

**Fig. 4:**
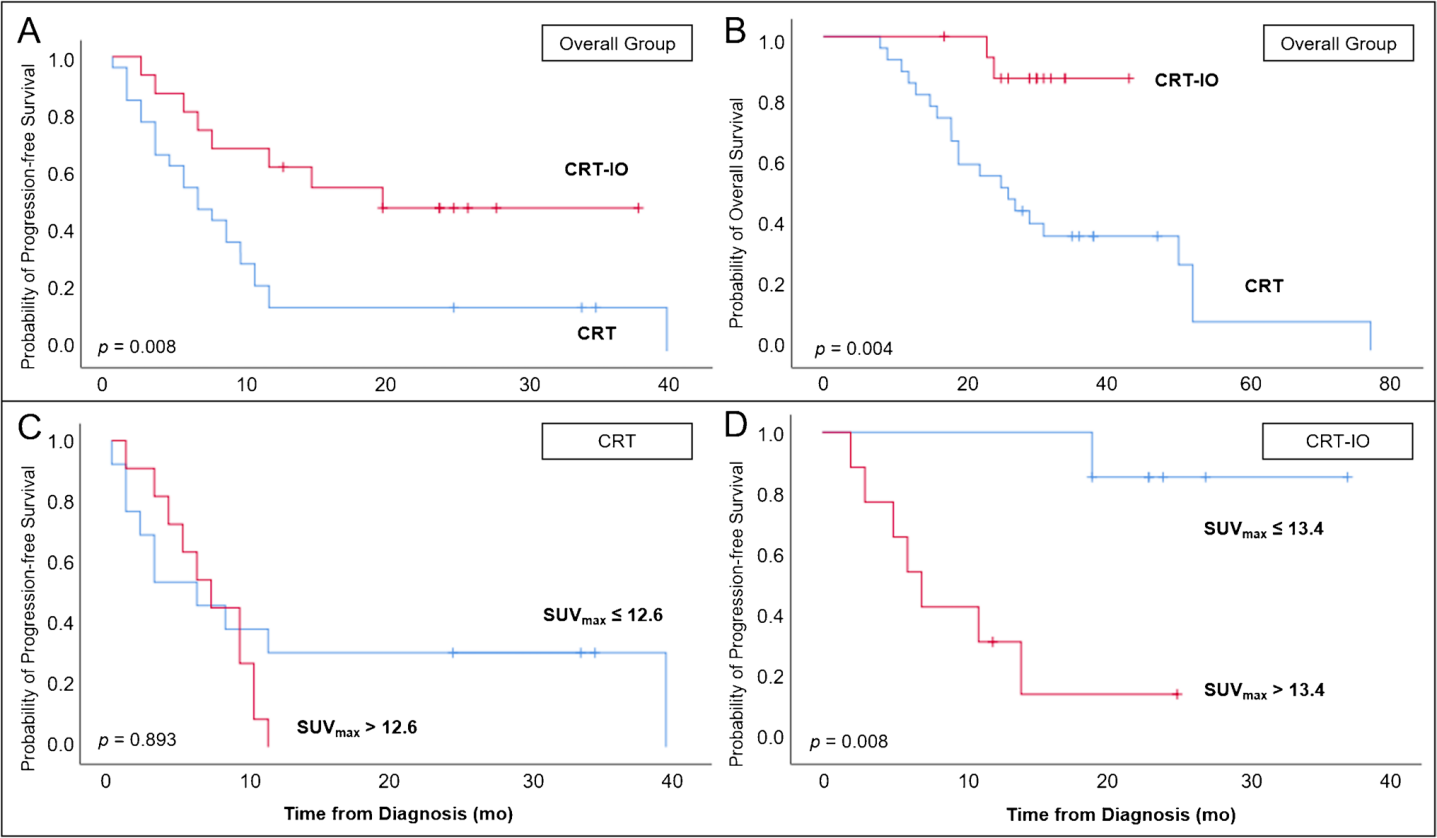
Progression-free (**A**) and overall (**B**) survival in patients undergoing chemoradiotherapy ± durvalumab maintenance treatment. In this cohort, a higher tumoral uptake on [^18^F]FDG PET/CT prior to chemoradiotherapy was not associated with survival in CRT (**C**) but with a shorter PFS in CRT-IO (**D**)

Median OS was 50.0 months (18.6 – 81.4), median PFS was 8.0 months (4.8 – 11.2). Patients treated with durvalumab maintenance treatment survived significantly longer without progression (19.0 vs. 6.0 months, *p* = 0.008; see Fig. 4A) and had a significantly longer overall survival (median not reached vs. 26.0 months, *p* = 0.004; see Fig. 4B) compared to patients undergoing chemoradiotherapy alone.

In line with the significantly prolonged survival in the CRT-IO group, the number of recorded clinical events was low: 8/16 patients experienced tumor progression and only 2/16 patients had died until the time of last follow-up.

In the overall group, a higher tumoral uptake on [^18^F]FDG PET/CT prior to chemoradiotherapy was associated with a shorter PFS (7 vs. 19 months, *p* = 0.021). In a subgroup analysis, the latter association was not present in patients undergoing CRT alone (*p* = 0.893; see Fig. 4C). However, in patients receiving durvalumab maintenance treatment, the tumoral uptake prior to chemoradiotherapy was associated with survival: A high tumoral uptake above SUV_max_ 13.4 was associated with a shorter PFS (*p* = 0.008; see Fig. 4D). None of the other imaging parameters assessed was significantly associated with survival (each *p* > 0.05).

## Discussion

There is only a paucity of reported data regarding [^18^F]FDG PET/CT findings in unresectable stage III NSCLC patients treated with CRT followed by durvalumab maintenance therapy. Here, we present the first preliminary data regarding metabolic uptake patterns on [^18^F]FDG PET/CT in unresectable stage III NSCLC patients receiving durvalumab maintenance treatment in comparison to patients undergoing CRT alone. Distinct metabolic changes were noted in tumoral lesions and secondary lymphoid organs. Further, administration of durvalumab was associated with a higher proportion of findings suggestive of immunotherapy related-adverse events.

In the current era of evolving immunotherapy with breakthrough successes in NSCLC, the role of accompanying accurate imaging is increasingly recognized and emphasized [20]. Promising work has focused on patient selection for immunotherapy and cutting edge tracers have been developed that directly address therapeutic targets such as PD-L1 in NSCLC [21–23]. However, an even greater clinical need is to gain a better understanding of how new immunotherapeutic treatments impact glucose metabolism as detected on [^18^F]FDG PET/CT: Decisions regarding therapy management in NSCLC commonly rely on interpretation of [^18^F]FDG PET/CT images, but may be hampered by immunotherapy-associated atypical imaging patterns [13, 24]. Whereas in other entities or with the use of different immune checkpoint inhibitors in NSCLC, altered imaging patterns have already been acknowledged [14, 25, 26], to our knowledge this is the first work to examine a distinct group of patients with unresectable stage III NSCLC treated with durvalumab maintenance therapy, which is the new standard of care in this cohort [3].

Diverging metabolic patterns were observed in secondary lymphoid organs of NSCLC patients dependent on durvalumab administration: The spleen uptake increased in the CRT-IO group as opposed to the CRT group (median + 12.5% vs. –4.4%, *p* = 0.029) and followed a trend of increasing spleen volume in the CRT-IO group (+ 14.1% vs. – 1.3% in the CRT alone group, *p* = 0.092). An increased spleen metabolism has been described as a surrogate of immune activation with the potential to stratify patients at an early time point, and spleen-related [^18^F]FDG uptake parameters on PET have been proposed as a prognosticator for patients under immunotherapy [27–29]. PD-1 is not only expressed on T cells being activated but also on B cells and even some non-hematopoietic cells, and the PD-1/PD-L1 axis interferes with multiples pathways of immune activation, thus explaining the increased spleen metabolism in the CRT-IO group [30, 31]. As the cohort was rather small and as few deaths were recorded due to substantially prolonged survival with durvalumab maintenance treatment, no subgroup analysis regarding immunotherapy response within the CRT-IO group could be performed. Studying melanoma patients, a significantly increased spleen metabolism was observed at 2 weeks in responders, while such a rise could not be observed in non-responders (+ 49% versus − 1%, p < 0.05), underscoring the potential value of an imaging-based surrogate parameter for immune activation which could enable a non-invasive early response prediction to immune checkpoint inhibition [32]. The highlighted value of the temporal aspect of metabolism in secondary lymphoid organs will be explored in NSCLC patients who receive durvalumab maintenance treatment, once more clinical events have occurred.

In line with a divergent glucose metabolism in secondary lymphoid organs as a sign of immune activation, a significantly higher proportion of findings suggestive of irAEs was noted in the CRT-IO group (75.0% vs. 29.6% of cases, p = 0.005). Pneumonitis-typical imaging findings were common in both groups, and pneumonitis is also known to occur as a direct sequela of irradiation independently from immunotherapy [33]. However, 50% of cases in the CRT-IO group showed imaging findings suggestive of a pneumonitis, which is remarkably higher compared to the previously reported prevalence of durvalumab-related pneumonitis and therefore indicates that [^18^F]FDG PET/CT might be a more sensitive approach to detect (potentially preceding clinically symptomatic) irAEs [34]. Only findings suggestive of reactive changes in the colon occurred more frequently in the CRT group, perhaps confounded by diabetes medication which can increase intestinal glucose metabolism [35]. All other findings suggestive of irAE including sarcoid-like reaction, thyroiditis, tonsillitis and vasculitis were more frequent compared to the CRT group or were observed exclusively in the CRT-IO group. An early or more sensitive detection of irAEs through [^18^F]FDG PET/CT could be clinically useful in two respects: First, early management of irAEs could reduce the rate and severity of complications [36]. Second, the occurrence of irAEs provides evidence to the treating physicians that the patient’s immune system has been successfully activated. As discussed above with regard to increased metabolism in secondary lymphoid organs, the occurrence of irAE could thus be an early, noninvasive predictor of response to immune checkpoint inhibition. However, whether an increased immune activation in terms of irAEs equates to increased antitumor immunity with a potentially improved survival outcome remains a controversial topic and needs to be elicited in future studies, also specifically regarding durvalumab maintenance treatment in NSCLC patients [37, 38].

Post-CRT uptake on [^18^F]FDG PET/CT has been shown to be associated with survival in locally advanced NSCLC [39]. Although an association of tumoral uptake with survival could be found in our cohort (a higher tumoral uptake on [^18^F]FDG PET/CT prior to chemoradiotherapy ± durvalumab was associated with a significantly shorter PFS), no statistically significant association with survival depending on the durvalumab administration could be found for post-treatment uptake or uptake changes over time. The possible reasons for the diverging association of a high tumoral uptake prior CRT with a short survival in between the two groups remain speculative. A study in patients with head and neck squamous cell carcinoma undergoing immunotherapy similarly revealed a lower [^18^F]FDG uptake on PET/CT prior to definitive therapy to be associated with a better clinical response and eventually linked this finding to the underlying mutational profile and hypoxia RNA signature [40]. In analogy, the initial pre-CRT level of [^18^F]FDG uptake found in our analysis might be a correlate for distinct inherent biological features of NSCLC predictive for the response to durvalumab. However, this hypothesis should not be over-emphasized due to the small number of patients in the CRT-IO group and the findings have to be confirmed in studies with larger data sets and hence higher statistical power. In general, the low number of clinical events recorded in the CRT-IO group, i.e. a low number of deaths, has probably hampered the identification of tumor-related imaging-based prognosticators in this cohort so far (only 12.5% of patients in the CRT-IO group died until the time of last follow-up). This is consistent with the groundbreaking survival benefit of durvalumab demonstrated in the PACIFIC trial, as recently confirmed in the updated five-year overall survival data with an estimated 42.9% of patients receiving durvalumab maintenance treatment after CRT remaining alive at 5 years [7]. Still, the tumoral uptake reduction was significantly higher in patients receiving durvalumab (SUV_max_ –70.0% vs. –24.8%, *p* = 0.009), allowing the assumption that post-treatment uptake may indeed be of prognostic relevance in the context of durvalumab in NSCLC. Survival analysis will be substantiated in correlation to metabolic changes as soon as more clinical events are present. Eventually, in this context, prospective correlational studies including imaging but also other modalities are needed to establish more reliable methods to characterize tumor response and control. A prospective longitudinal biomarker study evaluating immunological, molecular-genetic, image-based, and microbial analyses in patients with unresectable stage III NSCLC treated with concurrent chemoradiotherapy followed by durvalumab maintenance treatment is underway [41].

As an outlook, next steps may include a longitudinal investigation to establish a correlation of described immune-related surrogate imaging markers with peripheral expansion of corresponding T-cell subpopulations on several time points within durvalumab maintenance treatment, especially in PD-L1 tumor cells positive and negative patient cohorts. Further, an integration of durvalumab-specific hybrid imaging before and within durvalumab maintenance treatment will be of interest to clarify a predictive and prognostic role of PD-L1 positive durvalumab-avid tumor and immunocompetent cell populations [42, 43].

## Conclusion

Durvalumab maintenance treatment after CRT leads to diverging tumoral metabolic changes, but also increases splenic metabolism and leads to a higher proportion of findings suggestive of irAE compared to patients without durvalumab. Due to significantly prolonged survival in the CRT-IO group, survival analysis will be substantiated in correlation to metabolic changes as soon as more clinical events are present.

## Data Availability

The datasets generated during and/or analyzed during the current study are mainly given in the manuscript. Further data may be available from the corresponding author on reasonable request.
